# Inequality in households’ access to primary health care (PHC): a case study in Kerman, southeast Iran

**DOI:** 10.1186/s12913-022-08467-4

**Published:** 2022-08-23

**Authors:** Hamed Rahimi, Reza Goudarzi, Somayeh Noorihekmat, AliAkbar Haghdoost, Fatemeh Khodabandeh

**Affiliations:** 1 Students Research Committee, Kerman University of Medical Sciences, Kerman, Iran; 2 Health Services Management Research Center, Institute for Futures Studies in Health, Kerman University of Medical Sciences, Kerman, Iran; 3 Modeling in Health Research Center, Institute for Futures Studies in Health, Kerman University of Medical Sciences, Kerman, Iran; 4 Students Research Committee, School of Management and Medical Information Sciences, Shiraz University of Medical Sciences, Shiraz, Iran

**Keywords:** Access, Equity, Fair, Primary health care, Households, Concentration index

## Abstract

**Background:**

Fair access to health services is a vital issue in low-and middle-income countries. Therefore, the present study was conducted to evaluate the equity in access to primary health care (PHC) services in southeastern Iran.

**Methods:**

This household-based survey was conducted on 1128 households in Kerman, southeastern Iran in 2019-20. A multistage probability method was used to select the samples. The online questionnaire was designed and its link was provided to the questioners. After receiving the training, the questioners went to the door according to the sampling guide. The collected data were analyzed at a significance level of 0.05, using the STATA software. The concentration index (CI) was also used to measure inequality in access to PHC services.

**Results:**

The results showed that there was a significant difference between gender and location in access to PHC services (*P* < 0.05). However, no significant difference was found between the access rates to PHC services and the variables of age, marital, education, health insurance, and Supplementary insurance (*P* > 0.05). The mean rate of access to PHC services was 3.51 ± 0.53. Cultural access (3.76 ± 0.54) and timely receipt of PHC services (2.51 ± 0.72) accounted for the highest and the lowest access rates, respectively. The concentration index for the distribution of PHC services among the income-adjusted population was 0.014 (CI 95%: -0.022 to 0.051), indicating pro-rich inequalities in access to PHC services.

**Conclusion:**

The results indicated that pro-rich inequality, but it was close to the equality line. Also, the access level was assessed as moderate to high. Therefore, planning and policy-making seems essential for reduce inequality, and development and promotion of access to PHC services, especially timely provision of services and organizational access.

**Supplementary Information:**

The online version contains supplementary material available at 10.1186/s12913-022-08467-4.

## Background

Ensuring community health is a key element in the economic growth of countries. In contrast, increased burden of diseases slows it down [1]. This encourages governments to have strong health systems in order to improve the health of their communities [2, 3]. In this regard, World Health Organization (WHO) has considered two elements, including the highest access and equity, necessary to achieve the goals of health systems [4]. Besides, the Universal Health Coverage (UHC) Agenda has emphasized the right of all people to access essential, quality services when needed, without financial hardship. Equitable access to quality health services has also been the focus of sustainable development goals [5].

By Anderson’s definition, access refers to the actual use of health services and anything that facilitates or prevents the use of these the services [6]. Access to health services can also be defined as the degree of fit between clients and the health system [7]. Access to health services is critical to improving community health in low-and middle-income countries (LMICs), because knowledge and awareness of access to health services in these countries helps to plan for allocation of resources to different levels of the health system, monitor access to UHC, and ensure fair access to health services [8].

Equity is a key concept in access assessment, and includes equal benefit of the people with equal needs (Horizontal Equity) and lower benefit of those with fewer needs (Vertical Equity) [6, 9]. Thus, equitable access to primary health care (PHC) services as the first level of contact with health service consumers seems critical [10, 11], because evidence suggests that countries with strong PHC systems have better population health outcomes, reduced health inequity among demographic groups, and reduced avoidable hospitalizations [12–14]. However, while PHC policies have focused on reducing barriers to access [12], inequality in PHC access is on the rise [11].

Considering numerous challenges to accessing PHC, and given the fact that providing PHC services is associated with increased equity [15], assessing fair access to PHC is a basic step toward improvement. The information on access to health services is vital and helpful to health managers and policymakers [8]. Previous studies have shown that Iran’s PHC system faces challenges in the areas of manpower, service delivery and access [16–18]. In another study, reduced access is predicted as the consequence of social trends affecting the Iranian health system in the future [19]. It may cause inequity in accessing the PHC system.

 The Declaration of Alma-Ata was approved in 1984 by the Iranian government and parliament and led to the development of a health network. Over time, as the burden of diseases and the people’s health needs changed, some improvements were done in PHC [18]. Implementation of PHC is one of the country’s main strategies for achieving public health coverage and reducing the gap between health outcomes in rural and urban areas [20]. Iran provides PHC services within the framework of a health network system. The network is structured at three levels: national, provincial and city levels. At the national level, the Ministry of Health is responsible for policy making, planning and financing. At the provincial level, state universities of medical sciences plan and oversee the services. Each province has developed a health network at the county level. The network consists of two urban and rural centers managed by the city health center [16].

In the urban areas, comprehensive urban health centers and health posts are responsible for providing health services to the people directly, and in the rural areas, comprehensive rural health centers and health-houses do the job [21]. Health-house is the smallest unit of PHC system in rural areas that cover 1200 people [22]. Since 2005, the Family Physician Program has been implemented as a strategic intervention to develop the health system, increase access to and productivity of health care, and reduce inequalities in rural areas and less affluent (poor) cities [23, 24]. Besides, the urban family physician program is currently being implemented as a pilot project in Fars and Mazandaran provinces [23, 25]. In terms of financing, PHC in Iran is mainly funded and provided by the government [26]. So that PHC coverage in rural areas is reported to be more than 95% [27].

Given that no study has been conducted to examine the fairness of access to PHC in Iran, therefore the present study was conducted to evaluate the equity of access to PHC services in southeastern Iran.

## Methods

### Setting

This is a household-based survey conducted in southeastern Iran in 2019-20. The research population consisted of the households under the coverage of Kerman University of Medical Sciences (KMU), which is the largest university in Kerman province in southeastern Iran and covers nine cities with 399,783 households.

### Sample size and design

We used population mean formula to calculate sample size. According to the formula, Z = 1.96, standard deviation (s) = 0.5 and margin of error (d) = 0.035, sample size was calculated 1067 households. By considering a design effect of 1.4 and 10% non-response rate, the total sample size was 1176 households and finally 1128 households answered the questionnaire (response rate: 95.92%).

**Fig. 1:**
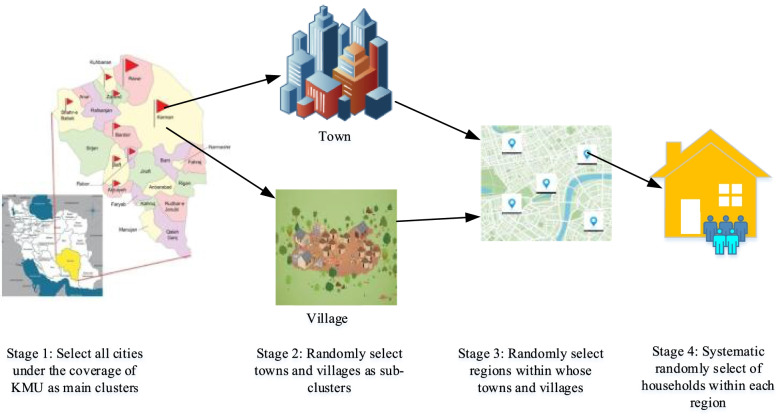
Multistage method of select the samples

A multistage probability method was used to select the samples. At first, the cities were considered as the main clusters. The number of samples in each cluster was calculated considering the number of households as a quota. Then, based on the national divisions by the Ministry of Interior, a number of towns and villages of each city were randomly selected as sub-clusters. Next, some regions in the urban and rural clusters were randomly selected as well. In the urban clusters, at least 2 and at most 8 urban areas were selected, depending on the number of samples. In the rural clusters, 2–3 villages were randomly selected based on the national divisions by the Ministry of Interior. Finally, the systematic random sampling method was used to select households (Fig. 1). In the urban areas, the data were collected from that samples and continued to one of each three houses to the right by specifying cluster heads (the first house on the right side of the street or alley). In the rural areas, the first house on the right of the village entrance was selected as the first sample and the data were collected from one of each three houses. If any sample was not present or did not respond, the next house was selected. Given the fact that some of the selected villages might have fewer inhabitants than the sample size or be uninhabited, several villages were reserved.

### Measurement instrument

The data collection tool was a self-made questionnaire to measure people’s access to PHC services. The questions were designed by the research team in collaboration with experts in the field of PHC. The validity of the questionnaire was assessed using the face and content validity. The face validity was examined and confirmed by 9 experts, and both qualitative and quantitative methods were used to determine the content validity. In the qualitative method, the questionnaire was reviewed by 30 health workers and people, and necessary changes were made. The Content Validity Ratio (CVR) and Content Validity Index (CVI) indices were then calculated for quantitative analysis. Thus, the questions with CVR > 0.78 and CVI > 0.79 ​​were accepted. The internal consistency method and the Cronbach’s alpha were also used to evaluate the reliability of the questionnaire. Before conducting the study, the questionnaire was completed by 80 randomly selected people. The Cronbach’s alpha coefficient of 0.82 was calculated. Thus, the questions were divided into two sections: demographic information (Mobile number, Gender, Age, Level of education, Marital status of the head of the household, Household income status, Place of residence (urban-rural), type of village (main, non-main), Health insurance status, Type of health insurance, and Supplementary insurance status) and access questions. The latter consisted of 49 questions in 6 dimensions, including geographical (3 item), financial (8 item), organizational (15 item), timeliness (4 item), cultural (14 item), and information (5 item) access. A 5-point Likert scale (5: very high, 4: high, 3: medium, 2: low, 1: very low) was used to answer the questions. Therefore, the minimum and maximum points were 49 and 245, respectively. Based on the mean score, access status was assessed in 3 categories: unfavorable access (0-1.5), middle access (1.5–3.5), and favorable access (3.5-5).

The household income level was asked of the head of the household. Households were divided into five income groups. According to the Central Bank of Iran, every dollar was considered equal to 42,000 Rials [28].

### Data collection

In order to collect the data, an online questionnaire was first designed and its link was provided to the questioners. After being trained, the questioners went to the houses to collect the data according to the sampling guideline. A verbal informed consent was obtained from those who were willing to participate in the study. Accordingly, they were assured that could withdraw at any time and their information would be used solely for the present study. The questions were answered by the head of the household or a member of the family over the age of 18 who had sufficient information and awareness. The specialized questions on women’s health were also asked of one of the women in the family. To ensure the accuracy of the data, the head of the household’s mobile phone number was taken and recorded. After collecting the data, the researcher randomly called a number of the samples and verified the data.

### Inequality indicators

The concentration index (CI) was also used to measure inequality in access to PHC services. The basis of the CI calculations is the Lorenz curve, and its values ​​vary from + 1 to -1. Negative values ​​indicated that the health variable was concentrated among the people with low income status, and the concentration curve was above the equality line. On the other hand, positive values ​​indicated the concentration of the health variable among the rich, and the concentration curve fell below the equality line. When the health variable distribution was the same among all individuals, the CI value was zero [29]. The relative CI was calculated using the following formula [9]:

$$\text{C}\text{I}=\frac{2}{{\upmu }}cov({h}_{i}, {R}_{i})$$

Where cov is the covariance, h stands for the health outcome (access), R is the rank of household i in the income distribution, and 𝜇 represents the mean access. We used STATA software v.12 for all calculations.

### Statistical analysis

The collected data were analyzed using the descriptive statistics and inferential tests at a significance level of 0.05. Firstly, the data normality was examined using the Kolmogorov-Smirnov test (*P*-value > 0.05). Considering the abnormality of some data, non-parametric Mann-Whitney and Kruskal-Wallis tests were used as well.

## Results

### Access

The results showed that most of the respondents were female (%52.5), aged 31–60 years (%71.3), married (%90.8), residents of urban areas (%53.7), having under-diploma education levels (%39.4), with basic insurance (%93.6), and with no supplemental insurance (%79.4). According to the statistical analysis, there were significant differences between the access rates of men and women and place of residence (*P* < 0.05). However, no significant difference was found between the access rates and age, marital status, education level, having basic and supplemental health insurance, and income level (*P* > 0.05) (Table 1).

**Table 1:** Access to PHC in KMU, Iran, 2019-20

Variable		N	%	Mean^*^	SD	*p*-value^**^
Sex	women	593	52.5	3.3	0.02	< 0.0001
	men	535	47.5	3.52	0.67	
Age	< 30 years	165	14.6	3.39	0.33	0.83
	31–60	804	71.3	3.41	0.33	
	60>	159	14.1	3.41	0.39	
Marital	Single	22	2	3.36	0.46	0.57
	Married	1024	90.8	3.4	0.33	
	divorced	12	1	3.3	0.35	
	death of parents	70	6.2	3.39	0.35	
Location	Urban	606	53.7	3.38	0.31	0. 011
	Rural	522	46.3	3.44	0.36	
Education	Illiterate	199	17.6	3.37	0.35	0.06
	High school	444	39.4	3.4	0.34	
	Diploma	360	31.9	3.4	0.33	
	Bachelor	98	8.7	3.44	0.64	
	Master & Ph.D.	27	2.4	3.5	0.85	
Health Insurance	Yes	1056	93.6	3.42	0.34	0.163
	No	72	6.4	3.27	0.29	
Supplementary Health Insurance	Yes	232	20.6	3.48	0.36	0.544
	No	896	79.4	3.39	0.33	
Income level ($)	> 1905	6	0.5	3.48	0.3	0.098
	1429–1905	13	1.2	3.6	0.32	
	953–1429	405	35.9	3.33	0.3	
	480–953	516	45.7	3.44	0.32	
	<480	188	16.7	3.46	0.43	

* From 5 score ** Significance level *P* < 0.05

The results showed that rural residents had more access to organizational, cultural, information and timeliness dimensions. According to the statistical analysis, there were a significant difference between rural and urban residents in terms of geographical, organizational, cultural and information access (*P* < 0.05). However, no significant difference was found between rural and urban areas in financial access and timely services (*P* > 0.05) (Table 2).

**Table 2:** Access dimensions to PHC by residence in KMU, Iran, 2019-20

Variable		N	%	Mean^*^	SD	*p*-value^**^
Geographical	Urban	606	53.7	3.77	0.87	< 0.0001
	Rural	522	46.3	3.48	0.81	
Financial	Urban	606	53.7	3.49	0.63	0.336
	Rural	522	46.3	3.48	0.62	
Organizational	Urban	606	53.7	3.17	0.38	< 0.0001
	Rural	522	46.3	3.38	0.48	
Cultural	Urban	606	53.7	3.73	0.4	< 0.0001
	Rural	522	46.3	3.83	0.46	
Information	Urban	606	53.7	3.48	0.61	< 0.0001
	Rural	522	46.3	3.81	0.63	
Timeliness	Urban	606	53.7	2.65	0.66	0.402
	Rural	522	46.3	2.66	0.62	

* From 5 score ** Significance level *P* < 0.05

**Fig. 2:**
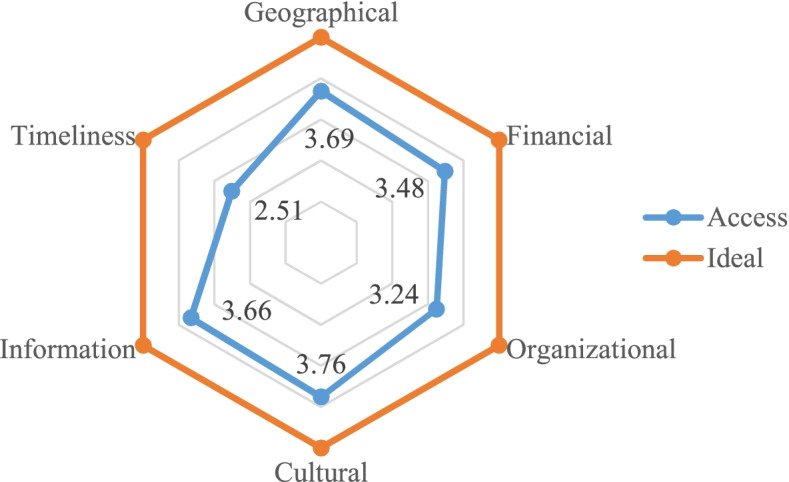
Level of access to PHC services in KMU, Iran in 2019-20

The results showed that the mean rate of public access to PHC services in KMU was 3.51 ± 0.53. Cultural access (3.76 ± 0.54) and timely receipt of PHC services (2.51 ± 0.72) accounted for the highest and the lowest access rates, respectively (Fig. 2).

### Concentration index

**Fig. 3:**
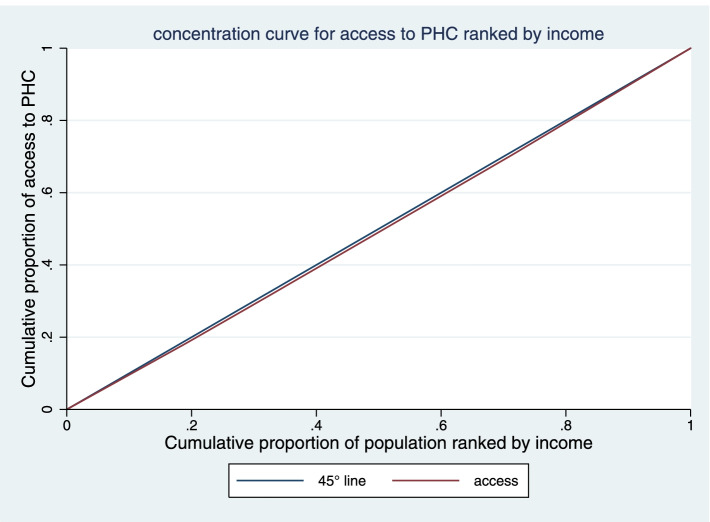
Concentration curve for access to PHC in KMU, Iran

Fair access of the income groups to PHC was examined using the CI. As shown in Table 1, the fourth (3.6 ± 0.32) and the third (3.33 ± 0.3) quintiles had the most and the least access to PHC, respectively. According to the CI analysis, the distribution of PHC access among the income groups was towards the rich in 2019-20. The CI of KMU in access of income-adjusted population to PHC was 0.014 (CI 95%: -0.022 to 0.051). As observed in the Fig. 3, the concentration curve was below the equality line, indicating pro-rich inequalities in access to PHC (Fig. 3).

**Fig. 4:**
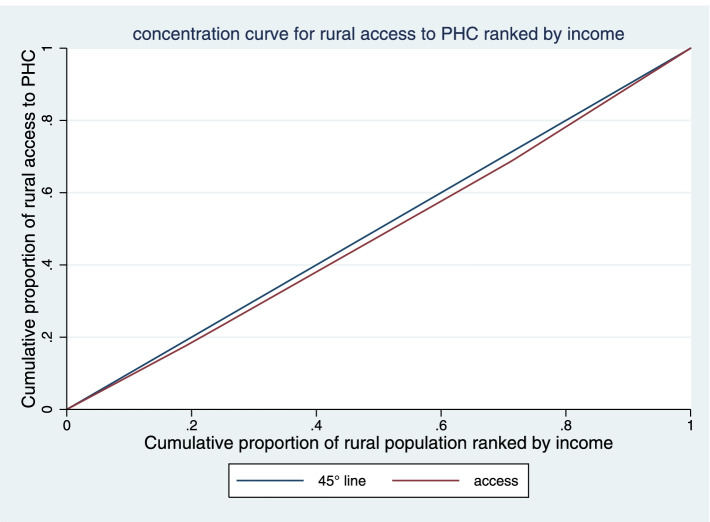
Concentration curve for access to PHC in rural areas of KMU, Iran

**Fig. 5:**
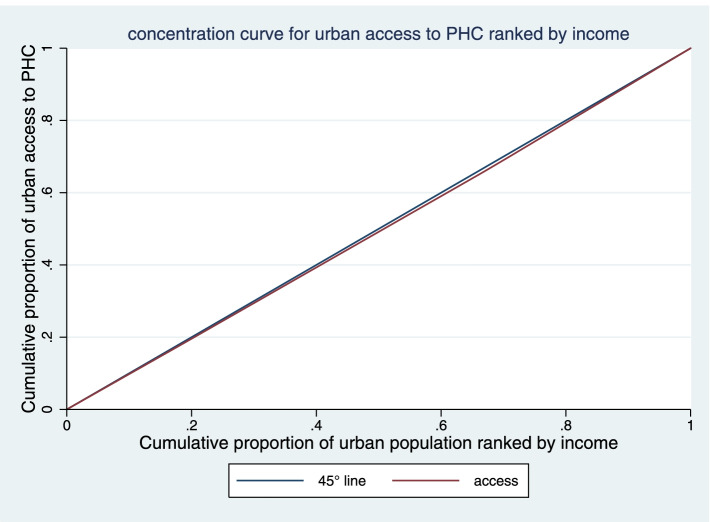
Concentration curve for access to PHC in urban areas of KMU, Iran

The CI of KMU in access of income-adjusted population to PHC in rural and urban areas were 0.031 (CI 95%: -0.001 to 0.064), and 0.011 (CI 95%: -0.0004 to 0.023), respectively. As observed in the Figs. 4 and 5, the concentration curve both in urban and rural areas was below the equality line, indicating pro-rich inequalities in access to PHC. But according to the results, there has been more inequality in rural areas.

## Discussion

Equity has always been an important issue in health policy-making and decision-making, and efforts to reduce health inequity is one of the priorities of countries and international organizations [30]. One aspect of inequity in health systems is people’s unfair access to health services, which has consequences such as unmet health needs, worse and unfair health outcomes, and higher healthcare costs [31]. Given that efficient and accurate evaluation is an essential and useful tool for health organizations and empowers the managers to control, monitor, and improve the quality of services and the performance of the organizations [32], this study evaluated the access level and equity of the Iranian PHC system.

Although the evaluation of the CI of access to PHC in Iran indicate pro-rich inequality, but it was close to the equality line. This indicated the lack of a big difference between the high- and low-income groups and it could be said that inequity in access to PHC was insignificant. Numerous factors could be involved in decreasing inequity in access to PHC, some of which were the cascading order of the Iranian PHC system’s structure, the implementation of the rural family physician program, Financing from public sources, some free PHC services provided, and the extensive coverage of the Health Insurance. Hassanzadeh et al. pointed out that one of the expectations from the implementation of the rural family physician program since 2005 and increasing health insurance coverage to over 90% was to increase access to and use of healthcare services by rural people and to reduce inequity [33]. Therefore, the results of this study showed that the policies to increase access and reduce inequity in the PHC system had been effective and efficient approximately. But it needs more effort for the remaining defects and problems.

The results of this study showed that the access level was reported to be moderate to high (3.5 of 5) and this needs to be improved. Rahimi et al. have listed access barriers as one of the challenges affecting the performance of Iran’s PHC [16]. A systematic review by Mehrolhassani et al. has reported geographic, cultural, and financial factors as challenges in accessing PHC services in Iran [21]. Another study found the PHC system in Iran to have challenges in governance, human resources, services delivery, technology, financing and information systems. They believe that the set of these challenges has weakened Iran’s PHC system and its current structure is not responsive to new needs, which has ultimately led to a decrease in people’s access to and use of PHC services [34].

Studying the access dimensions showed that the timeliness of the services was the poorest, with the mean score of 2.5 of 5. According to the results, only 8.3% of the households believed that they received timely PHC services, and 54.8% assessed it as poor. According to the results, the majority of the people received the services on the intended day but with a delay. It was found out that the biggest problem with receiving timely services was related to waiting for an appointment to see a doctor.

Delayed PHC services could cause physical and mental harm to the clients. On the contrary, providing timely services would lead to the people’s good experience of the healthcare delivery and would increase their satisfaction [35]. The results of a study by Papp et al. showed that people tended to have access to their general practitioners (GPs) as soon as they felt the need. However, they stated that people in different countries had different tolerances for waiting for GPs services. In Finland, for example, waiting for a week was acceptable for non-acute cases, but in other countries, such as Hungary, Lithuania, and Spain, any limiting waiting time was perceived negative are inappropriate [36]. Akinyinka et al. also estimated long waiting times in the Nigerian PHC system. They believed that reducing the waiting time could provide the basis for supporting PHC services and delivering health services cost-effectively [37].

Some studies have indicated that waiting time in acute conditions and diseases has a negative effect on the results of interventions. This is not true about non-emergency conditions and illnesses [38, 39]. Shortage of time in PHC provision is the biggest obstacle to evidence-based activities [40]. In addition, dissatisfaction due to long waits to receive government facilities may lead the individuals to the private sector [38], or in case of primary care, may lead to non-compliance with the referral levels and referring directly to specialized levels. Therefore, it is necessary to encourage people to use PHC services by avoiding long waits and providing timely services.

In general, various factors, both on the supply and demand sides, affect excessive waiting time, and this waiting time is mainly due to the imbalance between the supply and demand for health services. The factors such as high demand for health services, elderly population growth, technological advances, and physicians are influential in determining the amount of demand [38]. It should be noted that the waiting time is not always due to the problems on the supply side, but sometimes due to the demand side factors. Therefore, reducing the waiting time first requires rooting and accurately identifying the factors influencing the occurrence of waiting. Thus, it is recommended to consider this issue by managers and researchers in future studies.

On the other hand, in the situation where the waiting time is inevitable due to various reasons (such as high demand, time-consuming consultations, lack of doctors and other facilities, etc.), waiting conditions should be organized so as its destructive effects and consequences be reduced. Sherwin et al. believed that waiting time and space could be transformed from a waste of time into an opportunity. Using the waiting space for screening, monitoring, and training, which are the main functions of PHC, can enhance patient experience and lead to the provision of quality services, improved satisfaction, and efficient physician-patient counseling [41].

The results showed that from an organizational perspective, the access status was moderate and not favorable. One of the barriers to organizational access in the Iranian health system is the busy schedule and workload of PHC service providers. In their study, Bayati et al. also referred to the assignment of unrelated tasks to physicians. They believed that these tasks would increase family physicians’ responsibilities and workload and would have a negative impact on access to and quality of services [42]. Similarly, Amiresmaili et al. indicated that inadequate and long working hours as well as heavy workloads were an obstacle to providing services, which led to the withdrawal of GPs from the rural family physician program [43]. One of the consequences of poor organizational access is long waiting time that delays timely delivery of services [38]. Therefore, improving organizational access might lead to solving the problem of not providing timely services. One solution to reduce waiting time is to provide telephone consultations. Of course, physicians believe that telephone consultations require explicit guidelines. Having a telephone counseling guideline and reimbursement for such activities will increase professional satisfaction and patient safety, and will reduce waiting time [36]. increasing the PHC-provision facilities and services was a factor in improving organizational access to services and reducing the waiting time [44, 45].

Geographic access is one of the main aspects of equity in PHC. It is affected by distribution of PHC resources, especially the urban-rural distribution. The number of physicians should be determined based on the needs of the population. The rational distribution of activities and resources can facilitate access to primary care [36]. The distance dimension, i.e. the distance from residential houses to the health centers and the dispersion of villages in some areas, was the most important barrier to geographical access in the present study. Similar results were also obtained in the studies by Bagheri Lankarani and Eskandari, which confirm the results of the present study [46, 47]. Another study conducted by Shook on the transportation barrier and its role in accessing healthcare in one US state showed that two-thirds of the people had experienced transportation barriers and 40% of the people were unable to meet their health needs due to transportation barriers [48].

The results showed that access to PHC was higher in rural areas than in urban areas (Table 1). However, evidence suggests less access in rural areas [49–51]. The reason for the greater access of rural residents in this study may be more use of primary care services. In this regard, the study of Niyas et al. showed that in the villages there is more adherence to the referral system, perception of quality and the use of PHC services [52]. Also, in organizational, cultural and informational dimensions, villagers had more access to PHC than urban dwellers. One of the main reasons for the low level of organizational, cultural and information access in urban areas is the preference of people to use specialized and sub-specialized services and also the lack of attention of authorities to PHC services in urban areas. Niyas et al. Also believe that the primary care system has been abandoned in urban areas and the lack of attention of authorities has reduced their credibility [52]. However, in the geographical dimension, the urbanites had more access than the villagers to PHC. Researchers also believe that due to low population density and long distances villagers have to travel longer to access health centers, which reduces their geographical access [50, 53]. But higher access in rural areas has been accompanied by more inequity than the urban areas (Figs. 4 and 5). The WHO also believes that the core of the problem of inequity in primary care related to rural and remote areas [49]. Thomas et al. Point out that inequity access to PHC services in Australia is a factor in poor health for rural areas [54].

Given that equity in access to PHC is one of the social determinants of health, it is recognized as a strategy to eliminate health inequity [55]. The results of the present study showed that the Iranian health system had been able to develop a relatively fair primary care system. On the other hand, access to PHC is very important and necessary and has a significant impact on the people’s quality of life. Since access to PHC was found to be moderate to high, the following suggestions are provided to improve it: reducing the waiting time; strengthening access to home and work visitation services; education; improving the stability and durability of PHC providers; and ensuring proper access to all services, including diagnostic services required by the PHC system.

This study can increase the health system managers’ awareness of the current state of public access to PHC services and its equity. It can also help the health managers in policy-making and planning to improve access to such services. It is suggested that researchers conduct future family-based studies to examine access to PHC services and its equity in other provinces of Iran as well as other countries, especially LMICs.

The present study has some limitations. First, our sample was limited to one university medical sciences which may limit the generalizability of its results to other settings. Therefore, it is suggested that similar studies be conducted in other provinces and universities in the future. Second, Data about household income, experience of access and receipt of services are usually prone to recall bias. Third, higher / lower estimates of income are other limitations of such studies. Fourth, the close-to-zero CI in this study do not mean that the Iran’s PHC system does not experience access shortage. In fact, the CI is just indicate inequitable distribution of the access to PHC against economic gradient of the participants.

The strengths of this study include its large scale, its household-based nature, inclusion of both urban and rural areas, and having online questionnaires. The questionnaire system was designed in such a way that the questioners’ route and the place of completing the questionnaires could be traced. Furthermore, once the questionnaires were being completed, their output was provided to the research team. This could strengthen the validity and accuracy of the study data.

## Conclusion

The results of this study indicated that pro-rich inequality, but it was close to the equality line. Also, the public access level was assessed as moderate. Therefore, planning and policy-making seems essential for reduce inequality, and development and promotion of access to PHC system, especially timely provision of services and organizational access.

## Supplementary Information

**Additional file 1.**

## Data Availability

All data generated or analyzed during this study are included in this published article.

## Abbreviations

WHO: World Health Organization
UHC: Universal Health Coverage
PHC: Primary Health Care
LMICs: low-and middle-income countries
KMU: Kerman University of Medical Sciences
CVR: Content Validity Ratio
CVI: Content Validity Index
CI: Concentration Index
GPs: General practitioners
