# Supplementary material for: Inequality in households’ access to primary health care (PHC): a case study in Kerman, southeast Iran
Source: BMC Health Serv Res. 2022 Aug 23;22:1077. doi: 10.1186/s12913-022-08467-4 (PMC9400231; doi:10.1186/s12913-022-08467-4)
Supplement: Supplementary file 1 — Additional file 1. [file 12913_2022_8467_MOESM1_ESM.docx]

**Access to health services at the first level (PHC) Questionnaire**

Dear respondent, the present questionnaire that is presented to you in order to measure the extent of people's access to health services is the first level. Undoubtedly, your attention and accuracy in answering the questions will help us in providing information and obtaining useful results and providing valuable suggestions for improving health care. It should be noted that the information will remain strictly confidential and will only be used for research purposes. Your participation in the study is completely voluntary and you will be free to refuse to participate in the study or to leave the study whenever you wish.

Thank you in advance for your cooperation.

**A) Individual questions (head of household)**

**Gender:** male female **Age:** ……….

**Level of Education:** illiterate High school Diploma Masters Master and above

**Marital status:** Single Married Divorced Death of a spouse

**Address:** Rural Urban

**Family health insurance status:** Yes No

**Type of health insurance:** Health insurance Social Security Armed Forces Imam Relief Committee Other

**Supplementary health insurance status:** Yes No

**Household income:** ………………..

**Tel:** ...........................

**B) Access questions**

| **No.** | **Questions** |
| --- | --- |
| **Geographical access** | |
| 1 | To what extent is it possible for you to the health service centers in less than half an hour in the city or on foot in less than an hour in the village? |
| 2 | How accessible is the communication route to the health service centers throughout the year? |
| 3 | To what extent is there a convenient public transportation when you want to go from your home to health center? |
| **Financial access** | |
| 1 | Has it happened that you did not go to the health service centers because of the cost of receiving medicine? |
| 2 | Has it happened that you did not go to health service centers because of the cost of Para clinical services (imaging, laboratory)? |
| 3 | Has it happened that you did not go to health service centers because of the cost of midwifery consultation? |
| 4 | Has it happened that you did not go to health service centers because of the cost of Doctor visit? |
| 5 | Has it happened that you did not go to health service centers because of the cost of health and medical supplies? |
| 6 | Has it happened that you did not go to the health service centers because of the transportation cost? |
| 7 | Will receiving the required services from health service centers reduce your job income? |
| 8 | Have you ever had to borrow and sell living items and assets to go to health care centers and receive services? |
| **Organizational access** | |
| 1 | Do you refuse to go to the health service centers near your place of residence due to non-compliance with the principles of adaptation? (Such as the presence of many stairs, the lack of a suitable ramp) |
| 2 | Is it possible for you to talk privately with your doctor and other staff? |
| 3 | Are there adequate amenities such as chairs, benches, water coolers, and adequate waiting rooms in health care facilities? |
| 4 | Are there enough medicine and treatment items (Laboratory services, injections and dressings, sutures, midwifery services, vaccinations) in the health service centers? |
| 5 | Is cleanliness observed in the environment of your health service centers? |
| 6 | Do you have a shortage of manpower in health care centers relative to the population covered? |
| 7 | Did your family doctor age (Young or old) prevent you going to the center? |
| 8 | Is the time medical staff give you enough? |
| 9 | Does the center doctor have enough expertise and skills to treat your problem? |
| 10 | Are you satisfied with the services of other staff (except the doctor)? |
| 11 | Did you get it quickly in case of an urgent need for health care centers? |
| 12 | Were the working hours of the medical centers commensurate with the hours you could go? |
| 13 | When you are sick, does the doctor come to your home and visit you at home if you need to? |
| 14 | Is it possible for you to call the doctor when the health service centers are closed? |
| 15 | Do health service centers provide services on holidays and in the afternoons? |
| **Cultural access** | |
| 1 | Are the services of health centers provided to you according to cultural priorities? |
| 2 | Do health care providers overcome these barriers when they find out about your cultural differences? |
| 3 | When you visit new health care providers, Looking for information about your cultural needs? |
| 4 | Do health care providers identify potential cultural barriers that individuals may face? |
| 5 | Do health care providers pay attention to religion and other beliefs that may affect the response of individuals and families to health and illness? |
| 6 | Do you recommend the services of health centers to friends or relatives? |
| 7 | Do you recommend going to health centers for those who use traditional medicine (herbal or home remedies), or have specific beliefs about health care? |
| 8 | Have you ever wanted to go to a health center but did not go because your doctor was not your own gender? |
| 9 | Can immigrants (like Afghans) easily go to health centers? |
| 10 | Are the staff at your health service center native? |
| 11 | Do doctors and staff understand your local language and accent? |
| 12 | When you get sick, do you go to herbal and traditional medicine instead of the doctor in the first place? |
| 13 | Do you believe that you will get better when you see a family doctor? |
| 14 | Can women themselves go to health centers and use the services without anyone's permission? |
| **Information access** | |
| 1 | Are people at risk (Elderly, mothers, pregnant women, and children) informed about the location of health care centers and their services? |
| 2 | Do you know what services are provided in your comprehensive health service center? |
| 3 | Do you know the duties of a doctor? |
| 4 | Does your doctor give you clear explanations about the disease and the medications prescribed? |
| 5 | Have you been introduced to alternative centers for downtime and non-business hours? |
| **Timeliness** | |
| 1 | Were you able to receive the services you needed on the day you visited the center? |
| 2 | Is it possible for you to make an appointment in advance? |
| 3 | Do you have to wait to make an appointment at a health center? |
| 4 | Do you wait to see a doctor or other staff after attending a health center? |
